# Intervention and assessment of executive dysfunction in patients with stroke: A scoping review

**DOI:** 10.1371/journal.pone.0298000

**Published:** 2024-02-06

**Authors:** Katsuya Sakai, Yuichiro Hosoi, Junpei Tanabe

**Affiliations:** 1 Faculty of Health Science, Tokyo Metropolitan University, Tokyo, Japan; 2 Department of Rehabilitation of Medicine, Keio University School of Medicine, Tokyo, Japan; 3 Department of Sports Health Sciences, Ritsumeikan University, Kyoto, Japan; 4 Department of Physical Therapy, Hiroshima Cosmopolitan University, Hiroshima, Japan; Royal College of Surgeons in Ireland, IRELAND

## Abstract

Rehabilitation methods for executive dysfunction were focused on cognitive rehabilitation in patients with stroke and traumatic brain injury. However, no reviews have focused on the various rehabilitation methods and assessment of executive function in patients with only stroke and included various study designs. This study aimed to identify various interventions and assessments in patients with stroke and executive dysfunction via a scoping review. We searched for articles using the PubMed, Web of Science, and CINAHL databases. Two reviewers independently screened the articles based on the inclusion and exclusion criteria using the title, abstract, and full text. We subsequently determined the study design, sample size, time since stroke, intervention, and assessment. We extracted 1131 articles, of which 27 articles were selected. The study designs were randomized controlled trials (81.5%), pilot studies (11.1%), and feasibility studies (7.4%), with a total of 599 participants. Interventions varied from cognitive training (22.2%), virtual reality (22.2%), noninvasive brain stimulation (14.8%), and dual-task training (11.1%), with consistent results. The assessments used were the Trail Making Test Part B (70.4%), Stroop Color and Word Test (44.4%), Digit Symbol Test, Frontal Assessment Battery, and Tower of London test (11.1%). In conclusion, this scoping review provided various interventions and assessments in patients with stroke with executive dysfunction.

## Introduction

Executive function includes decision-making, risk-taking, planning, inhibitory control, working memory, and cognitive flexibility (speed, error processing, and attention) and is considered a higher brain function [1]. Despite it having various definitions [2,3], executive function is defined as a set of supervisory cognitive processes that integrates cognitive functions and supports the monitoring and regulation (task setting) of goal-directed behaviors [4]. Furthermore, the frontal lobes control these functions [5,6]; and impairment of other brain regions besides the frontal lobe and the widely distributed white matter results in executive dysfunction [5–8]. Approximately 25%–75% of stroke patients were reported to experience executive dysfunction, although this was related to the definition and assessment used [9,10]. Executive dysfunction is considered a silent epidemic because the impairment rarely manifests without assessment [9].

Executive dysfunction is associated with physical function and the ability to perform activities of daily living (ADL) and return to work [11–16]. Executive dysfunction is associated with walking ability in patients with stroke. Heyes et al. found a relationship between executive dysfunction and other walking tests, including a 10-m walking test, in patients with stroke [11]. Additionally, Sakai et al. reported that the degree of executive dysfunction was associated with performance on the Timed Up and Go test and a 10-m walking test in patients with stroke [12]. Lipskaya-Velikovsky et al. found a correlation between executive dysfunction and ADL in patients with stroke [14]. Moreover, Ownsworth et al. found that patients with stroke and executive dysfunction were less likely to return to work than patients with stroke without executive dysfunction [16]. Thus, executive dysfunction affects physical function and social reintegration.

Rehabilitation is necessary to improve executive dysfunction and facilitate the patient’s reintegration into society. Several rehabilitation methods have been previously reported in systematic reviews [17,18]. Cognitive training, such as working memory training, strategy training, and external compensatory approaches, improved executive dysfunction in patients with traumatic brain injury (TBI) and patients with stroke [17,18]. Working memory training include performing auditory and visual tasks using a computer, and verbal working memory training include memory tasks using alphabets [17]. The strategy training included goal management training and a computer assignment that solved ADL or instrumental ADL problems [17]. The external compensatory approach included training with a paging system to aid memory and planning [17]. Outcomes of executive dysfunction in patients with TBI and stroke were assessed using the Executive Function Performance Test and Multiple Errands Test [17]. Thus, assessment of the effects of rehabilitation on executive dysfunction focused on cognitive rehabilitation, demonstrating varied effectiveness. In addition, these reviews included patients with TBI and with stroke, and the number of studies was limited [17,18]. Furthermore, these systematic reviews included limited types of study designs and lacked assessment and rehabilitation methods for patients with only stroke [17,18]. Reviews on the various rehabilitation methods and assessments focused on patients with only stroke are lacking and no consensus on intervention methods and assessments, despite executive dysfunction being highly prevalent [9,10] and having a considerable effect on patients’ return to society [14,16]. Therefore, there is a need to identify and map the ways that have been previously overlooked in which executive dysfunction is assessed and treated in patients with stroke and to include a variety of study designs. After this, these factors should be investigated using methods such as systematic reviews. By focusing only on patients with stroke and mapping out assessment and rehabilitation methods for executive dysfunction including various study designs, this review aimed to fill the knowledge gap regarding rehabilitation for executive dysfunction and to provide useful evidence. Therefore, through this scoping review, the purpose of this study was to identify articles that had not been found in previous systematic reviews and to map and organize the knowledge of various interventions and assessments for executive dysfunction in patients with stroke.

## Methods

This scoping review followed the Preferred Reporting Items for Systematic Reviews and Meta-Analyses (PRISMA) Statement [19] and reporting guidelines by Arksey et al. [20] and Levac et al. [21]. This study was conducted in five stages (stage 1, identifying the research question; stage 2, identifying relevant studies; stage 3, study selection; stage 4, charting the data; stage 5, collating, summarizing, and reporting the results), following the work of Arksey et al. [20] and Levac et al. [21]. Data charting and summarization were performed using an analytic framework. Three databases (PubMed, Web of Science, and CINAHL) were used to search for articles from database inception to December 12, 2023. The following search terms were used in PubMed: (“Stroke”[MeSH Terms] OR “Hemiplegia”[MeSH Terms] OR “Hemiparesis”[MeSH Terms]) AND (“Executive Function”[MeSH Terms] OR “Executive Function”[All Fields] OR “Executive dysfunction”[All Fields]) AND (“Rehabilitation”[MeSH Terms]). The following search terms were used in the Web of Science databases and CINAHL: (Stroke OR Hemiplegia OR Hemiparesis) AND (Executive function OR Executive dysfunction) AND Rehabilitation. All articles were imported via EndNote (Clarivate, London, UK), and duplicate articles were removed and exported into Microsoft Excel (Microsoft Corp., Redmond, WA, USA).

Two reviewers independently reviewed the titles, abstracts, and full texts based on the inclusion and exclusion criteria and recorded article acceptance or rejection in independent Excel files. Each independent file was then combined into a single file, the results of the two reviews were combined, and papers with discrepant results were extracted. Subsequently, a third reviewer reviewed disagreements and accepted or rejected the papers. The inclusion criteria were as follows: 1) presence of stroke, 2) age > 18 years, 3) written in English, 4) randomized controlled trials (RCTs), feasibility studies, and case series, and 5) assessments of executive function. The exclusion criteria were as follows: 1) absence of stroke; 2) age < 18 years; 3) not written in English; 4) the use of non-human animals; 5) editorials, commentaries, conference abstracts, letters, reviews, books, case reports, and cross-sectional studies; 6) inclusion of immediate effects of interventions; and 7) inclusion of intervention details and assessment not yet described. Subsequently, Excel was used to chart each study’s author names, study design, participant number, time since stroke, intervention description, intervention period, assessment, and intervention results, taken from the full texts, after screening.

## Results

**Fig 1:**
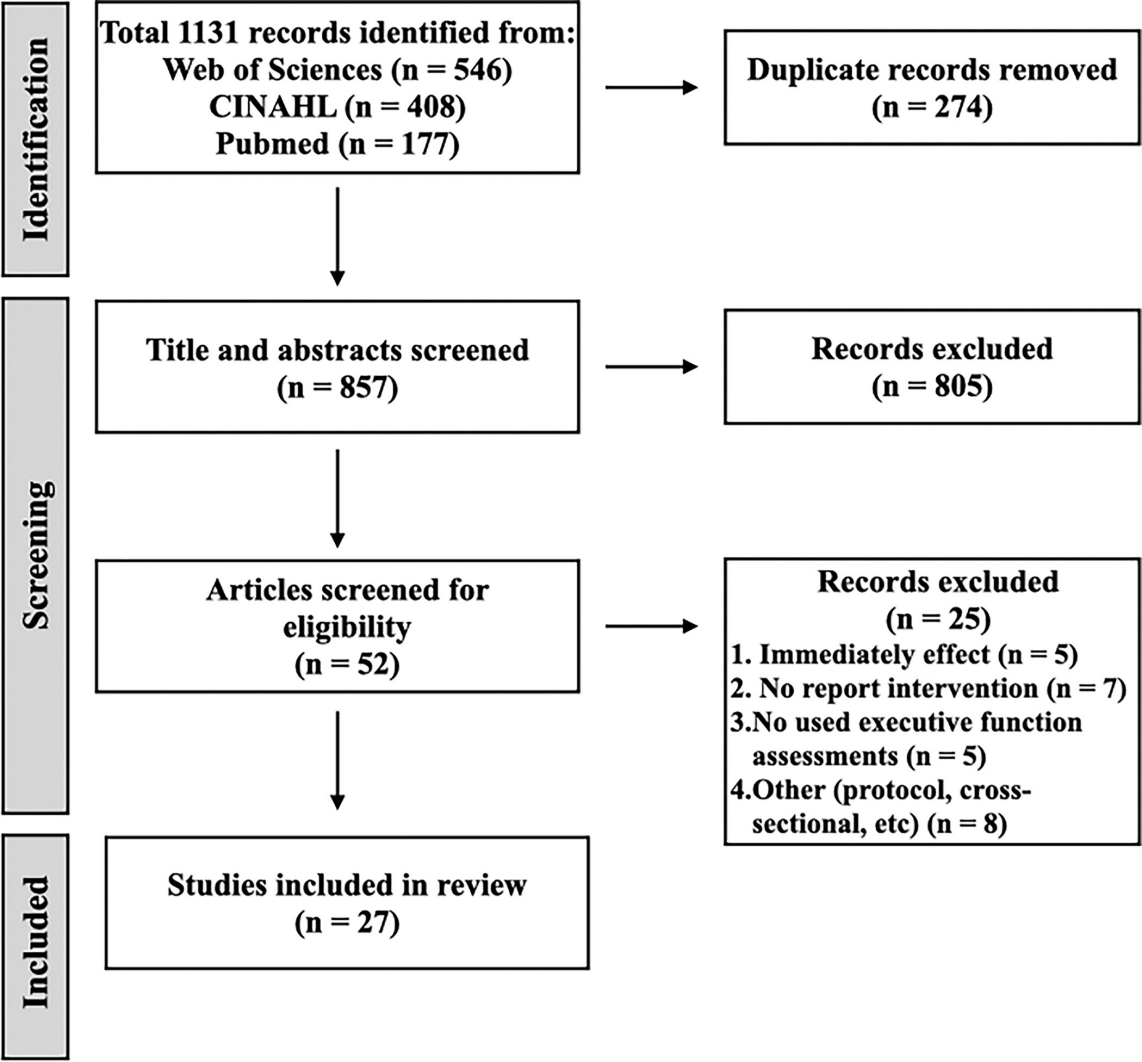
Preferred reporting items for systematic reviews and meta-analyses diagram for scoping review identification.

Fig 1 depicts the review process in the PRISMA diagram. From the three databases, 1131 articles were extracted; after removing duplicates, 857 articles were selected for primary screening. Following the inclusion/exclusion criteria described above, 52 papers were subjected to secondary screening and 25 papers were excluded. The reason for exclusion was that many articles did not provide detailed descriptions of the interventions or assessments. Finally, 27 papers were eligible for mapping. Table 1 indicates the results of the 27 selected articles. The rehabilitation methods newly identified in this study were virtual reality (VR), noninvasive brain stimulation (NIBS), and dual-task (DT) training with the Trail Making Test (TMT) Part B being the most commonly used assessment, followed by the Stroop Color and Word Test (SCWT).

**Table 1:** Results of mapping.

Authors	Design	Sample Size	Time since stroke	Intervention	Intervention Period	Outcome	Results
An H-S & Kim D-K, 2021 [22]	RCT	30	4 month	Dual task training	5 times/week, 5 weeks	TMT Part B, EFPT	The experimental group improved significantly compared to the control group.
Faria AL, et al., 2016 [23]	RCT	18	4–7 month	Virtual Reality	4–6 week	TMT Part B	There was no significant improvement after training compared to before training.
Gjellesvik TI, et al., 2020 [24]	RCT	70	25 month– 5 years	High-intensity interval training	3 times/week, 8 weeks	TMT Part B	The experimental group improved significantly compared to the control group.
Kim BR, et al., 2010 [25]	RCT	18	70–404 day	Repetitive transcranial magnetic stimulation	5 times/week, 2 weeks	SCWT, Tower of London test	There was no significant improvement after training compared to before training.
Lee S, et al., 2022 [26]	RCT	20	3–4 month	Virtual Reality with transcranial direct current stimulation	5 times/week, 4 weeks	TMT Part B, SCWT	The experimental group improved significantly compared to the control group.
Lin ZC, et al., 2014 [27]	RCT	34	227–228 day	Cognitive training	1 time/week, 10 weeks	TMT Part B	There was no significant improvement after training compared to before training.
Liu YW, et al., 2021 [28]	RCT	54	9 month	Transcranial direct current stimulation	5 sessions/time, 4 weeks	SCWT, DST	The experimental group improved significantly compared to the control group.
Liu ZL, et al. 2022 [29]	RCT	30	29–43 month	Virtual Reality	6 sessions/week, 6 weeks	DST	There was no significant improvement in the experimental group compared to the control group.
Liu-Ambrose and Eng JJ, 2015 [30]	RCT	28	3–4 month	Fitness and Mobility Exercise	2 sessions/week, 3–6 months	TMT Part B, SCWT	There was no significant improvement in the experimental group compared to the control group.
Maier M, et al., 2020 [31]	RCT	38	2.4–2.7 year	Virtual Reality	5 times/week, 6 weeks	TMT Part B, DST, FAB, Lastly	The experimental group improved significantly compared to the control group.
Manuli A, et al., 2020 [32]	RCT	90	6 month	Robotic rehabilitation with virtual reality	5 times/week, 8 weeks	TMT Part B, FAB	The experimental group improved significantly compared to the control group.
Park MO, et al., 2018 [33]	RCT	30	6 month	Cognitive-motor dual-task training with auditory motor synchronization training	6 weeks	TMT Part B, SCWT	The experimental group improved significantly compared to the control group.
Park MO, et al., 2019 [34]	RCT	30	21 month	Cognitive-motor dual-task training	3 sessions/week, 6 weeks	TMT Part B, SCWT	The experimental group improved significantly compared to the control group.
Poulin V, et al., 2017 [35]	RCT	30	5–11 month	Cognitive Orientation to daily Occupational Performance approach	2 sessions/week, 8 weeks	TMT Part B, SCWT	The experimental group improved significantly compared to the control group.
Rozental-Iluz C, et al., 2016 [36]	RCT	30	3.1–3.7 year	Interactive video-game intervention	2 sessions/week, 3 months	TMT Part B, EFPT	The experimental group improved significantly compared to the control group.
Tarantino V, et al., 2021 [37]	RCT	30	20–21 day	Executive function training	15 days	WCST, Five point test, SCWT	There was no significant improvement in the experimental group compared to the control group.
Torrisi M, et al., 2021 [38]	RCT	30	8.5–9 month	Virtual Reality with robotics hand training	5 times/week, 8 weeks	FAB	The experimental group improved significantly compared to the control group.
Van de Ven RM, et al., 2017 [39]	RCT	97	28–29 month	Computer-based cognitive flexibility training	5 times/week, 12 weeks	DEX	There was no significant improvement in the experimental group compared to the control group.
Yu H, et al., 2022 [40]	RCT	18	1 month	Repetitive transcranial magnetic stimulation	5 days/week, 2 weeks	SCWT	The experimental group improved significantly compared to the control group.
Bo W, et al., 2018 [41]	RCT	225	Unknown	Combined intervention of physical exercise and cognitive training	3 days/week, 12 weeks	TMT Part B, SCWT	The experimental group improved significantly compared to the control group.
Fernandez-Gonzalo R, et al., 2016 [42]	RCT	32	3.5–4.9 year	Flywheel resistance training	2 days/week, 12 weeks	TMT Part B, SCWT	The experimental group improved significantly compared to the control group.
Pallesen H, et al., 2019 [43]	RCT	38	1–3 month	The high-intensity training	4 weeks	TMT Part B, Tower of London test	The experimental group improved significantly compared to the control group.
Aprile I, et al., 2020 [44]	Pilot study	51	74.6 day	Robotics training	5 times/week, 1 week	SCWT, Tower of London test	There was significant improvement after training compared to before training.
Eschweiler M, et al., 2021 [45]	Pilot study	29	6 day	Cognitive and motor training	14 days	TMT Part B	The experimental group improved significantly compared to the control group.
Mark VW, et al., 2006 [46]	Pilot study	29	56.4–103.7 day	Constraint-induced movement Therapy	7 times/week, 3 weeks	TMT Part B	There was no significant improvement after training compared to before training.
Burdea GC, et al., 2020 [47]	Feasibility study	7	9 month	Telerehabilitation	5 sessions/week, 4 weeks	TMT Part B	There was no significant improvement after training compared to before training.
Huber SK, et al., 2021 [48]	Feasibility study	10	34 month	Personalized Motor-Cognitive Exergame Training	2 times/week, 8 weeks	TMT Part B, GO-NO GO test	There was no significant improvement after training compared to before training.

RCT: Randomized controlled trial, TMT: Trail Making Test, EFPT: Executive Function Performance Test, SCWT: Stroop Color and Word Test, DST: Digit Symbol Test, FAB: Frontal Assessment Battery, DEX: Dysexecutive Questionnaires.

### Study design, sample size, and stroke phase

Of the 27 articles, 22 (81.5%) [22–43], 3 (11.1%) [44–46], and 2 (7.4%) [47,48] articles were RCTs, pilot studies, and feasibility studies, respectively. The range of the sample sizes was 7–225 participants (mean 22.2 ± 13.0), with 599 participants in total. The range of time since stroke was 6 d to 5 years. Three (11.1%) [37,40,45], four, 13, and four articles included the acute (< 1 month), subacute (18.5%) [22,26,30,43,44], chronic (48.1%) [24,27–29,31–34,36,38,39,42,47,48], and subacute to chronic (14.8%) phases [23,25,35,46], respectively.

### Interventions

Interventions were cognitive rehabilitation (6/27 articles, 22.2%) [27,35,39,45,47,48], VR (6/27 articles, 22.2%) [23,26,29,31,32,38], NIBS (4/27 articles, 14.8%) [25,26,28,40], DT training (3/27 articles, 11.1%) [22,33,34], and other various training methods (8/27 articles, 29.6%) [24,30,37,41–44,46]. The intervention period was at least twice a week, and the duration varied from 2 weeks to 3 months and was not consistent. Cognitive rehabilitation included executive function, memory, and working memory training using computers [27,35,39,45,47,48]. VR was used in a variety of ways, including tasks to practice the components of each executive and other (memory, decision making) function, systems to solve tasks through games, and systems to carry out training based on ADL [23,26,29,31,32,38]. Studies using NIBS used repetitive transcranial magnetic stimulation (rTMS) and transcranial direct current stimulation (tDCS) to target the prefrontal cortex, but the parameters varied [25,26,28,40]. DT training was performed while walking, calculating, avoiding obstacles commonly occurring in daily life, and climbing stairs [22,33,34]. Although the intervention content was divided into several parts (cognitive rehabilitation, VR, NIBS, DT training), there was no consistent rehabilitation method. Other training included Robotics training [44], Constraint-induced movement Therapy [46], and various training [24,30,37,41–43].

### Assessments

Assessments included the TMT Part B (19/27 articles, 70.4%) [22–24,26,27,30–36,41–43,45–48], SCWT (12/27, 44.4%) [25,26,28,30,33–35,37,40–42,44], Digit Symbol Test (3/27, 11.1%) [28,29,31], Frontal Assessment Battery (FAB) (3/27, 11.1%) [31,32,38], Tower of London Test (3/27, 11.1%) [25,43,44], Executive Function Performance Test (2/27, 7.4%) [22,36], and other assessments (i.e., Dysexecutive Questionnaire and Five-point Test) (4/27, 14.8%) [31,37,39,48].

## Discussion

The purpose of this study was to identify articles that had not been found in previous systematic reviews and to map and organize the knowledge on various interventions and assessments for executive dysfunction in patients with stroke. We found 27 articles in three databases. We found several RCTs (81.5%), in which the stroke phase was mostly chronic (48.1%). The interventions were cognitive training (22.2%), VR (22.2%), NIBS (14.8%), and DT training (11.1%). The assessments were the TMT Part B (70.4%), SCWT (44.4%), Digit Symbol Test, and FAB, Tower of London Test (11.1%), and Executive Function Performance Test (7.4%). VR, NIBS, and DT training were the first rehabilitation methods extracted in this study. In addition, TMT Part B was the most commonly used assessment, and SCWT was also frequently used, and these assessments were also extracted for the first time in this study. This review provided various interventions and assessments in patients with stroke and executive dysfunction.

### Interventions

The most common intervention was cognitive rehabilitation and VR (22.2%), followed by NIBS (14.8%), and DT training (11.1%). Cognitive rehabilitation was common, as described in a previous systematic review (17,18). This scoping review extracted, for the first time, the articles related to VR and NIBS, which have recently been used in several rehabilitation areas [49–56].

Cognitive rehabilitation involves various methods, and the cognitive training mapped in this review was traditional training similar to that described in previous studies [17,18]. Typical examples include executive and working memory training with personal computers [27,35,39,45,47] to solve problems using various modalities, including attention, memory, executive function, and vision [27,39,45,47]. Others included strategy training, including goal setting, execution, and evaluation to determine their achievements [35]. Previous systematic reviews that included patients with stroke and TBI and cognitive rehabilitation were extracted [17,18]. The rate of cognitive rehabilitation was high, although only patients with stroke were included, similarly to those in this review. Therefore, cognitive rehabilitation may be more common in the treatment of executive dysfunction.

In this scoping review, VR training for executive dysfunction in patients with stroke was extracted for the first time, including six articles (22.2%) [23,26,29,31,32,38]. Simple VR training included traditional computer-based cognitive rehabilitation methods implemented using VR [31]. Additionally, other VR training methods included patients visiting places they frequently went to in their daily lives in a simulated space to solve tasks (e.g., going to a supermarket to shop) [23] or patients solving tasks in a game [29,38]. Hence, the training content related more to daily life and real-life situations than cognitive rehabilitation.

Additionally, articles that used NIBS were extracted for the first time in this scoping review, which included four articles (14.8%) [25,26,28,40]. NIBS included tDCS [28], rTMS [25,40], and NIBS in conjunction with VR or traditional cognitive training [25,26,29,40]. We included a study in which 2–mA tDCS was applied to the prefrontal cortex for 20 min, with simultaneous cognitive training [28]. Additionally, we included studies in which rTMS was provided, with differences in stimulus frequency and each parameter, such as high and low frequency stimulation [25,40]. However, the stimulation region was commonly the prefrontal cortex [25,40]. The combined tDCS and VR study used stimulation of 20 min at 2–mA; however, the stimulation site was the primary motor cortex, and the VR was used to perform a motor task [26]. The articles extracted in this review used prefrontal cortex stimulation, sharing common stimulation areas, but NIBS has different effects depending on the stimulation areas and parameters. Therefore, examining various parameters is necessary in the future.

Additionally, three articles using DT training (11.1%) were extracted. DT training is performed while walking, calculating, avoiding obstacles common in daily life, and climbing stairs [22,34]. Additional training to add auditory stimuli to DTs, such as during calculations, was also reported [33]. DT training is more dynamic than other training methods (cognitive rehabilitation, VR, and NIBS) owing to its involvement of calculations and obstacle avoidance while walking. Daily life often involves DT situations.

### Assessments

The TMT Part B was the most frequently used assessment (70.4%), followed by the SCWT (44.4%). In a previous systematic review of patients with only stroke, the TMT Part B was used in 3 of the 10 included articles (30%) [17]^.^ The other seven articles were inconsistent [17]. The TMT Part B can be used to assess executive dysfunction, including attention, memory, sequencing, decision-making, automatic thinking, set-shifting, and cognitive flexibility [57–59]. We assumed that the TMT Part B would be used at a high rate because of its ease in determining intervention effectiveness compared with the method used in a previous review [17]. The SCWT can also be used to rapidly and easily assess executive function, including attention, processing speed, cognitive flexibility, and working memory [60,61]. However, the TMT Part B and SCWT include only limited aspects of executive dysfunction when compared with the FAB, the Behavioral Assessment of Dysexecutive Syndrome, and other assessments. Contrastingly, the FAB classifies executive function into six items and facilitates a rapid and easy assessment; however, it was used in 11.1% of the 27 included articles. This may be because the FAB is used in several studies involving elderly patients and patients with dementia and Alzheimer’s disease [62,63]. However, the FAB may be used as an assessment method when examining the effectiveness of each component because it can easily evaluate each component (i.e., conceptualization, intellectual flexibility, motor programs, sensitivity to interfering stimuli, inhibition, non-influence) of execution function. However, given that the FAB has a maximum score of 18 points with 0–3 points for each item, the ceiling and floor effects must be considered.

### Future recommendations for research and interventions

This scoping review mapped existing cognitive rehabilitation as well as previously unreported rehabilitation methods such as NIBS and VR. This included studies that had been overlooked in previous systematic reviews. However, the interventions varied, the study designs were not uniform, and no established rehabilitation methods could be identified. NIBS (rTMS, tDCS) is expensive compared to VR, and VR is becoming increasingly available and inexpensive. Therefore, VR may be easier to implement in clinical rehabilitation settings. In addition, DT training is a method that could be reasonably adapted to patients without requiring special devices. The TMT Part B and SCWT were often used for assessment, but these can only capture a portion of executive dysfunction. However, we consider these easy-to-use evaluation tools because of their short evaluation time, high sensitivity compared with the FAB and Tower of London Test, and potential to sensitively reflect treatment effects. Therefore, future research in this area should consider study designs, conduct effectiveness testing of various rehabilitation methods, and use rating scales to assess various aspects.

### Strengths and limitations

This scoping review focused on patients with stroke and various rehabilitation methods, using a widespread strict search of electronic databases (from RCTs to case series). This restricted the search to patients with stroke and provided an evaluation of intervention types and assessments that had previously been overlooked. However, this scoping review had some limitations. A clear definition of executive dysfunction was lacking, which may have resulted in some relevant articles being inadvertently omitted due to a difference in the terms being used (i.e., cognitive impairment). Additionally, executive function could not be itemized. Therefore, future research should define executive function using components and identify interventions and assessments, including their effects. Finally, the quality of the studies was not assessed in this study. This is because the quality assessment of studies in scoping reviews is not performed in the guidelines [20]. However, other previous guideline has indicated that a quality assessment of the study should be considered [2]. The current study includes five papers for which no quality assessment other than RCTs is possible, which would make it impossible to map the various assessments and intervention methods for executive dysfunction, which is the aim of this study. In addition, a systematic review is planned as the next step. Therefore, this study did not choose to assessment the quality of the study. However, in the case of scoping reviews that focus on intervention content, results, and parameters, study quality assessment should be considered.

### Conclusions

In this scoping review, VR, NIBS, and DT training were extracted as rehabilitation methods. Additionally, TMT and SCWT were the most common assessments that determined the effectiveness of the intervention. However, the extracted articles varied in intervention duration, intensity settings, and protocols, and it was not possible to reach a consensus on intervention content. Therefore, future research should be conducted based on these factors. This review provided various interventions and assessments in patients with stroke and executive dysfunction.

## Supporting information

S1 Checklist Preferred Reporting Items for Systematic reviews and Meta-Analyses extension for Scoping Reviews (PRISMA-ScR) checklist. (PDF)

S1 File (XLSX)
